# Total knee arthroplasty according to the original knee phenotypes with kinematic alignment surgical technique—early clinical and functional outcomes

**DOI:** 10.1186/s12891-020-03862-6

**Published:** 2020-12-11

**Authors:** Cheng-En Hsu, Jen-Ting Huang, Kwok-Man Tong, Kui-Chou Huang

**Affiliations:** 1 Sports Recreation and Health Management Continuing Studies-Bachelor’s Degree Completion Program, Tunghai University, Taichung, 407 Taiwan; 2 Department of Orthopedics, Taichung Veterans General Hospital, Taichung, Taiwan; 3 Department of Orthopedics Surgery, Asia University Hospital, 222 Fuxin Rd., Wufeng District, Taichung City, 41354 Taiwan; 4 Department of Occupational Therapy, Asia University, Taichung, Taiwan

**Keywords:** Kinematical alignment, Kinematically aligned; Total knee arthroplasty, TKA, TKR, Posterior stabilization, Phenotype of the knee, Total knee replacement

## Abstract

**Background:**

The kinematic alignment (KA) technique in total knee arthroplasty (TKA) aims to restore the native alignment of pre-disease knee joint anatomy. Determining the individualized alignment targets is crucial for pre-operative planning, which can be set according to different original knee phenotypes. Five most common knee phenotypes have been categorized for KA-TKA alignment target setting in our previous study. The purpose of this study was to investigate the distribution of the five phenotypes in advanced OA knee patients and evaluate the clinical outcomes of this phenotype-oriented KA-TKA using the generic instrument, with particular emphasis on alignment strategy, surgical technique, survivorship, radiographic and functional outcomes.

**Methods:**

The clinical data of 123 patients (88 women, 35 men) who had undergone 140 TKAs in our hospital were reviewed. All the TKAs were performed with alignment targets set according to the original phenotypes of the knee, with the KA method, using the generic total knee instrument. The patients’ demographics, preoperative and postoperative knee alignment angles, one-year postoperative range of motion (ROM), Oxford knee scores (OKS), Combined knee society score (CKSS) were collected and analyzed.

**Results:**

The 3 years survivorship was 99.3% for all cause of revision, and 100% with revision other than infection as the endpoint. The preoperative phenotypes of the knee were as follows: neutral alignment 20.1% (type 1: 3.6%, type 2: 16.5%), varus alignment 71.2% (type 3: 46.0%, type 4: 25.2%), and valgus alignment (type 5: 8.6%). Using our protocol, patients with different knee phenotypes could get similar great functional improvement though the postoperative alignment parameters were significantly different between the knee phenotypes (*P* < 0.05).

**Conclusion:**

The early outcomes of this phenotype-oriented KA-TKA using generic total knee instruments are promising. Setting individualized alignment target according to original knee phenotype is rational and practical. The residual varus alignment did not cause any aseptic loosening in the 3 years follow-up. Long-term survivorship and functional outcomes need to be evaluated in future studies.

## Background

Though total knee arthroplasty is the definitive surgical treatment for advanced osteoarthritis of knee (OA knee), high dissatisfaction rates of up to 20% are still reported in uncomplicated TKA [1–3]. This high dissatisfaction rate has raised the question as to whether the aim of bone resection should be to achieve ideal mechanical alignment that is appropriate for each patient.

In contrast to the mechanical alignment (MA) method which aims to create a neutral alignment by cutting the tibia and femur perpendicular to the mechanical axis [4, 5], the kinematic alignment (KA) method aims to restore the pre-disease knee joint by resecting bone parallel to the pre-disease joint line of the femur and tibia [6]. This method attempts to decrease the anatomical change of bone, thus potentially minimizing the impact on ligament balance. Recently a randomized-control study indicated that KA-TKA led to significant better improvement in quantitative knee balance than MA-TKA [7]. A 10-year follow-up study also showed that KA-TKA had excellent implant survival, yearly revision rate and function level [8]. However, two problems are often encountered in KA-TKA operation. First, in patients with very advanced osteoarthritis, it is difficult to make a precise estimation of wearing thickness of bone. Second, in patients with severe malalignment in lower extremities. Placing prosthesis in mal-aligned axis may compromise the long-term survival.

To achieve more individualized alignment for KA-TKA, the distribution of knee alignment among patient populations has been investigated and the most common five phenotypes have been categorized for the alignment target setting in our previous study [9]. In our protocol, more individualized alignment targets were set for each patient according to the each patient’s knee phenotype. The KA-TKAs were then performed according to the preoperative alignment targets. The purpose of this study was to investigate the distribution of the five phenotypes in advanced OA knee patients and evaluate the functional and radiographic outcomes of this phenotype-oriented KA-TKA method.

## Material and methods

### Patient enrollment

This study is a prospective series. The medical and radiographic records of patients with advanced OA knee who received TKA in our hospital between August 1st, 2015 to October 31th, 2016 were collected prospectively.

The inclusion criteria were patients with mental health that could signed the informed consent form and completed all the functional status questionnaires and measurements of this study. The exclusion criteria were patients undergone simultaneous bilateral TKA and revised TKA.

### Data collection

**Fig. 1:**
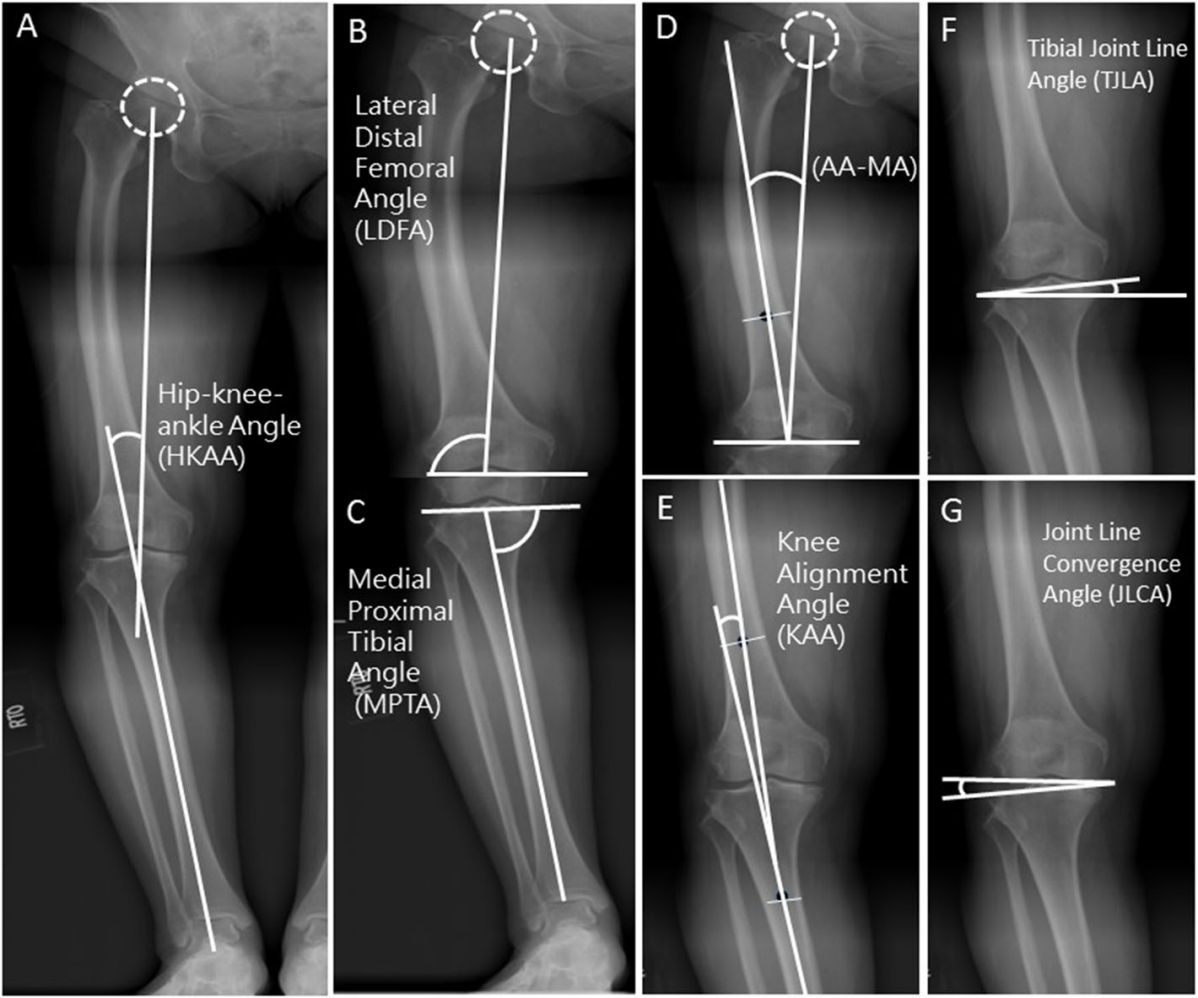
Measured Coronal Knee Alignment Angles. The five angles were defined as the following: **a** The Hip–knee–ankle angle (HKAA): the angle between the mechanical axis of the femur and the tibia. The value of HKAA was defined as positive in varus alignment. **b** Lateral distal femoral angle (LDFA): the lateral angle between the mechanical axis of the femur and the distal femur joint line, which is defined as the connection of the lowest points of the medial and lateral femoral condyle. **c** Medial proximal tibial angle (MPTA): the medial angle between the mechanical axis of the tibia and the proximal tibia joint line, which is defined as the connection of the lowest points of the medial and lateral tibial plateau. **d** Angle between the femoral anatomical axis and the mechanical axis (AA-MA): the angle between the mechanical axis and the anatomical axis of the femur. **e** Knee alignment angle (KAA): the angle between the anatomical axis of the femur and the anatomical axis of the tibia in the short film of the knee. The value of KAA was defined as positive in varus alignment and as negative in valgus alignment. **f** Tibial joint line angle (TJLA): the angle formed by the parallel line to the floor and the proximal tibia joint line. If the two lines intersect with an angle on the lateral side of the leg, it is a medial open angle. If two lines intersect with an angle on the medial side of the leg, it is a lateral open angle. Lateral open angle is presented as a positive value, medial open angles as a negative angle. **g** Joint Line Convergence Angle (JLCA): the angle between the knee joint lines of the distal femur and proximal tibia

All angles of lower extremity alignment were measured on the long leg radiograph. The hip-knee-ankle angle (HKAA), knee alignment angle (KAA), tibial joint line obliquity angle (TJLA), lateral distal femur angle (LDFA), medial proximal tibial angle (MPTA), angle between the mechanical axis of femur and anatomical axis (AA) were measured preoperatively [9] (Fig. 1). All measurements were performed independently by two blinded observers using the GeoGebra 5.0 software (International GeoGebra Institute, Austria, 2016). When the measurement value was different, the revised one was determined after re-measurement and discussion by the two observers.

The phenotypes of the knee were categorized mainly according to the difference of mechanical alignment of the femur and the tibia (LDFA and MPTA), as described below:

1. The mechanical alignment of the femur was divided into varus, neutral, and valgus alignment. Varus was defined as LDFA≧90°, neutral as 87° ≤ LDFA < 90°and valgus as LDFA< 87° [9].
2. The mechanical alignment of the tibia was defined as varus, neutral and valgus alignment of the tibia. Varus was defined as MPTA < 87° and neutral as 90° > MPTA≧87° and valgus as MPTA≧90°.

**Fig. 2:**
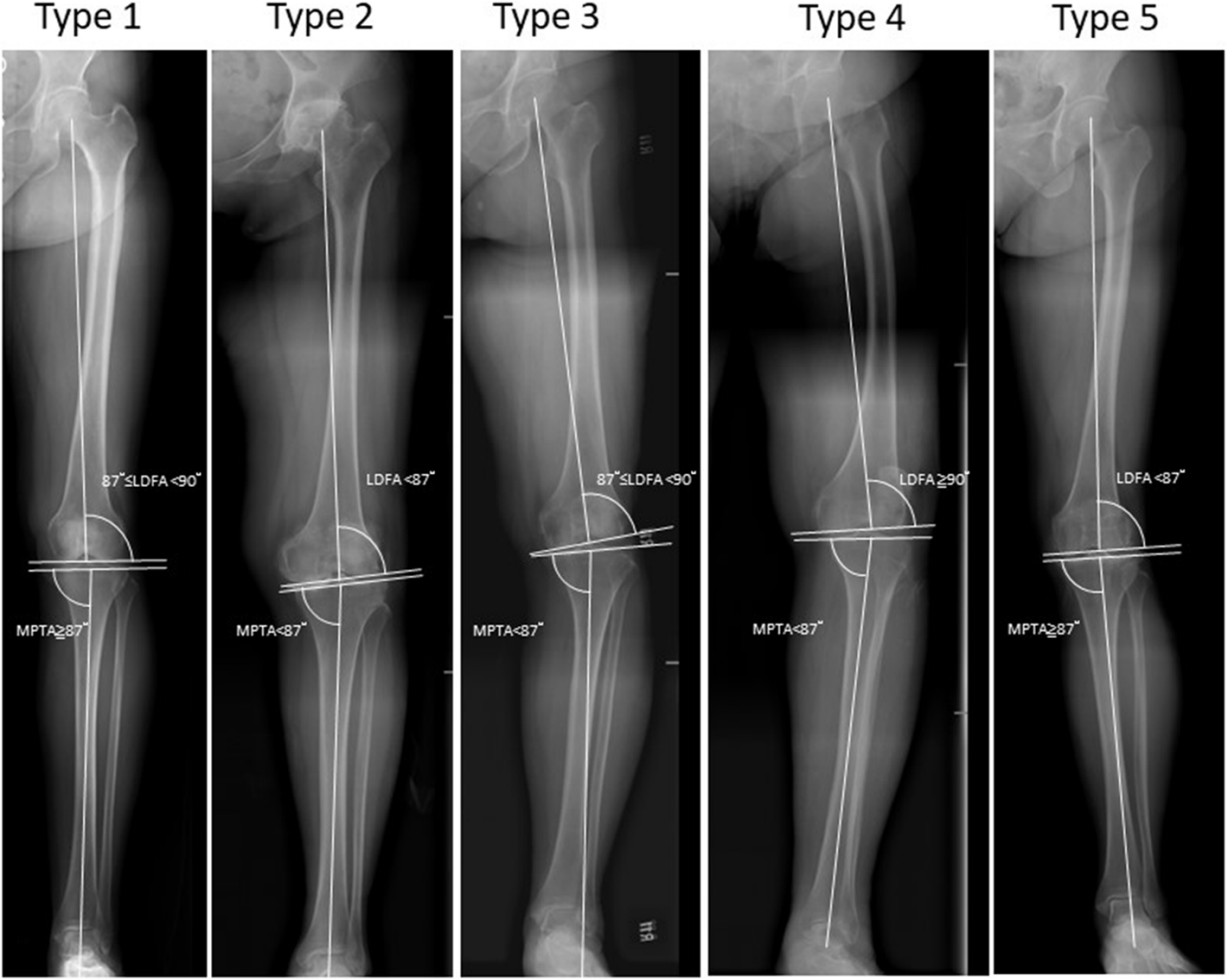
The five most common knee phenotypes categorized according to the difference of mechanical alignment of the femur and the tibia (LDFA and MPTA)

Based on the above different alignments of tibia and femur, five most common knee phenotypes could be categorized (Fig. 2), as described by our previous study [9]: Type 1 knee had neutral alignments in lower limb, the femur and the tibia (Mean HKAA: 0.6°, LDFA 88.0°, MPTA 87.0°). Type 2 knee also had a neutral alignment of the lower limb, but a high degree of joint obliquity of the knee (Mean HKAA: -0.4°, LDFA 85.0°, MPTA 85.1°). Type 3 knee had a varus alignment of the lower limb, but the distal femur remained valgus or neutral (Mean HKAA 4.2°, LDFA 88.0°, MPTA 83.5 °). The main cause of varus alignment of the lower limb was due to proximal tibia varus alignment. Type 4 knee had concomitant varus alignment of the tibia and femur (Mean HKAA 5.6°, LDFA 91.4°, MPTA 85.2°). A high degree of varus lower limb alignment and lateral bowing of the femur are usually observed in this type of knee. Type 5 knee had a valgus alignment of the lower limb with femur exhibiting a valgus alignment, but the tibia had a neutral or valgus alignment (Mean HKAA: -4.2°, LDFA 84.6°, MPTA 88.8°). Patients with valgus tibia alignment (MPTA≧90°) were included in type 5 knee for their number was very small and the operation method was similar to type 5 knee [9]. The lower limb alignment was valgus, which was mainly contributed by the femur.

We set different alignment targets for LDFA and MPTA according to each original phenotype of the knee. The preoperative planning, surgical technique, pain management, and rehabilitation program were as follows.

### Preoperative planning

**Fig. 3:**
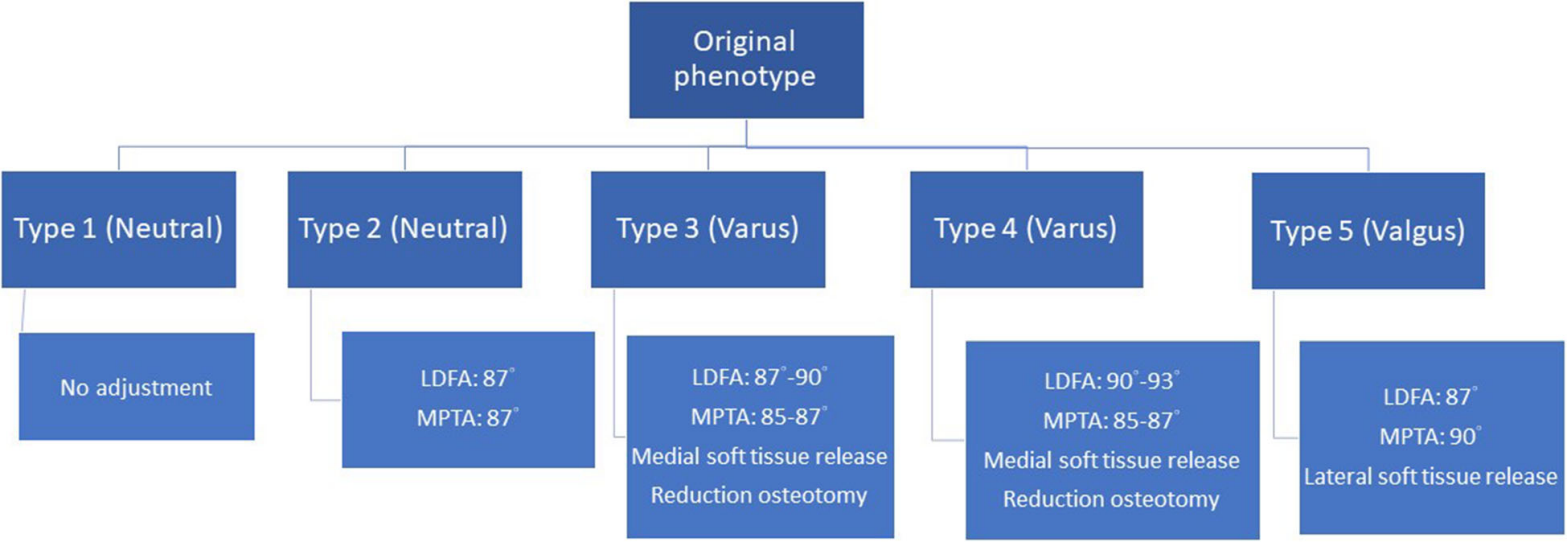
The algorithm for target angles of LDFA and MPTA according to original phenotype of knee

We first determined the original phenotype of the knee according to the alignment of the tibia and femur as described above. For type 1 knee, which has a neutral alignment and transverse joint line, we cut the distal femur and proximal tibia parallel to the original joint line. No adjustment of alignment was made. For type 2 knee, which is characterized by a high oblique joint line, we adjusted the LDFA and MPTA 2–3° to decrease the joint line obliquity. The target of LDFA and MPTA was set at 87°. For type 3 knee, which has a high degree tibia varus, we cut the distal femur according to the original joint line, and the target of tibia alignment adjustment for MPTA was 85–87°. For type 4 knee, which is characterized by a concomitant varus tibia and femur, the femur usually has lateral bowing with LDFA> 95°. We adjusted the LDFA to 90–93° and the MPTA to 85–87° to correct the varus alignment of the lower limb. For type 5 knee, which has a valgus femur, the target of LDFA was set at 87°, and the target of MPTA was set at 90°. The targets of alignment adjustment for each phenotype are described in Fig. 3.

### Intraoperative surgical technique

**Fig. 4:**
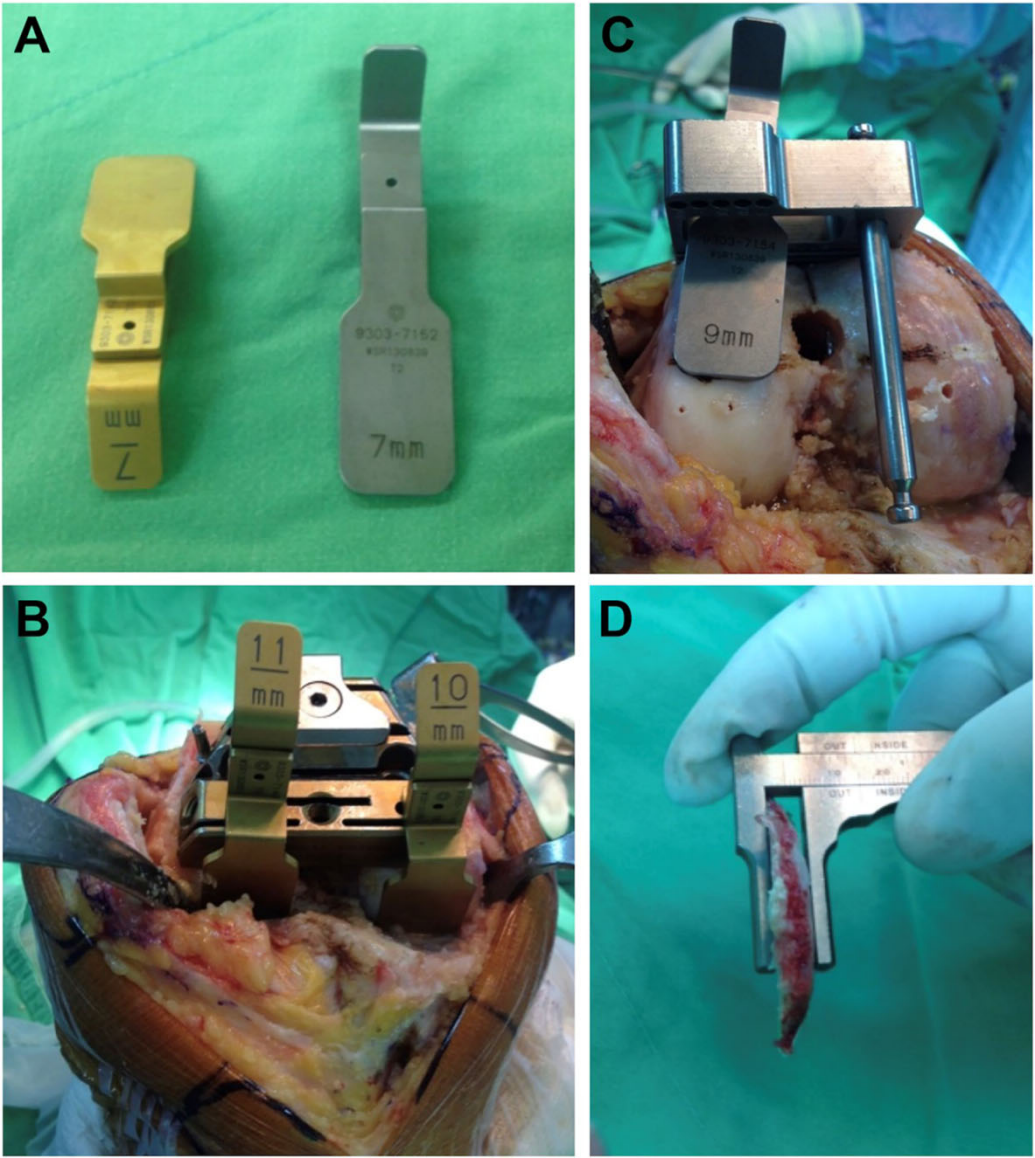
Measure resection of distal and posterior femur condyle: **a** Distal and posterior femur cutting guide, **b** Distal femur cutting guide. **c** Posterior femur cutting guide, **d** Caliper to check the thickness of bone cut

All surgeries were performed by a team lead by single experienced surgeon utilizing a posterior stabilized knee (Zimmer Biomet, LPS flex, Warsaw Indiana, USA) using midline skin incision with a medial parapatellar arthrotomy. After removal of all the osteophytes, the superficial and deep medial collateral ligaments were released for better exposure. The distal femur was first resected to achieve the target LDFA. A designed cutting guide was used to evaluate the thickness of the distal femur cut (Fig. 4). In a varus knee, the ideal distal femur cut should be 7 mm and 9 mm for the medial and lateral sides, respectively. If adjustment of the distal femur cut is made, each 1° change requires a cut of 0.65–0.7 mm [10]. The thickness of bone cut should be measured again until the target range is achieved. Then, the knee was flexed to 90°. The femoral axial rotation was set parallel to the preoperative-planned MPTA target. A designed cutting guide was used to evaluate the thickness of posterior femur cut. In a varus knee, the ideal posterior femur cut should be 11 mm and 10 mm for the medial and lateral sides, respectively. Each 1 millimeter adjustment can be estimated to correspond to 1.2°-1.5° rotational change of the femoral axis [10]. The posterior femur cut should be 2 mm less than the thickness of the prosthesis to reduce the flexion instability (the posterior thickness of a Zimmer LPS flex is 12 mm), as the release of the posterior cruciate ligament increases the flexion gap by 2-3 mm [11]. Femoral component sizing was measured using a posterior condyle referencing device. After cutting the femur bone, the proximal tibia was cut according to the target MPTA with 5° of posterior slope. Trial implants were placed, and soft tissue releases were performed to create balanced extension and flexion gaps. A balanced gap is usually achieved with subperiosteal release of superficial and deep medial collateral ligament (MCL), transverse, and longitudinal incision of the posteriomedial capsule without release of pes anserinus and pie crust of the deep MCL.

In a valgus knee, the distal femur bone was cut to form an LDFA of 87°. The proximal tibia was cut with a jig perpendicular to the mechanical axis of the tibia, posterior slope 5°, such that the MPTA was 90°. Then the knee was placed in the extended position, and a trial insert was placed. Release of the iliotibial band, arcuate ligament, and lateral posterior capsule were performed to balance the extension gap. Then the knee was flexed to 90°, the femoral axial rotation was set parallel to the tibial plateau, which was cut perpendicular to the tibial mechanical axis. For a valgus knee, the ideal posterior femur cut should be 11 mm and 8 mm for the medial and lateral sides, respectively.

### Post-operative evaluation

Patients were followed-up at 2 weeks, 1 month, 3 months, 6 months, 1 year, and 2 year after operation. Postoperative HKAA, LDFA, MPTA, KAA, and TJLA were measured in a long leg radiograph at 6 months follow-up. ROM, OKS and CKSS were recorded at 12 months after the operation.

### Statistical analysis

Data plotting and statistics were processed using the GraphPad Prism software for Mac OS X. Values represent the mean ± SD. All data were processed for Gaussian distribution with D’Agostino & Pearson normality test initially. The patients’ demographics, knee alignment angles (HKAA, KAA, TLJA, MPTA, LDFA), and functional knee scores (OKS, CKSS, ROM) were assessed with the Wilcoxon matched-pair signed rank test and one-way ANOVA. The level of significance was set at *p* < 0.05.

## Results

A total of 123 patients (140 knees) received the operation during the study period. One patient with diabetes who encountered prosthesis infection received two-stage revisional TKA was excluded. A total of 122 patients (139 knees) were included in the final analysis. The 3-year survival rate was 99.3% for all-cause revision and was 100% for revision other than infection. No aseptic loosening or instability was observed.

The demographics, preoperative and postoperative knee alignment angles and functional scores of the 122 patients are shown in Table 1. Their mean follow-up time was 36.5 months (31 to 42). The preoperative lower limb alignment distribution of the 139 knees were 28 (20.1%) with neutral alignment, 99(71.2%) with varus alignment, and 12 (8.6%) with valgus alignment. Significant improvement was observed in HKAA, KAA, TJLA, MPTA, OKS, CKSS, and ROM after the operation.

**Table 1:** Demographics and radiographic findings of 122 patients treated with phenotype-oriented KA-TKA

	Pre-operative data	Post-operative data
Male: Female (*n* = 122)	35:87	35:87
Age (years±S.D)	70.5 ± 7.08	70.5 ± 7.08
Left: Right (*n* = 139)	76:63	76:63
HKAA (° ± S.D)	8.9 ± 8.50	3.4 ± 3.64^****^
AA (° ± S.D)	6.9 ± 2.09	
KAA (° ± S.D)	0.6 ± 6.4	−4.5 ± 2.6^****^
TJLA (° ± S.D)	2.4 ± 3.42	1.0 ± 1.80^****^
LDFA (° ± S.D)	88.4 ± 3.15	88.1 ± 2.14 (*p* = 0.069)
MPTA (° ± S.D)	83.3 ± 3.53	85.2 ± 2.47^****^
OKS (Points ± S.D)	17.6 ± 7.66	44.0 ± 3.31^****^
KSFS (Points ± S.D)	90.2 ± 30.59	172.1 ± 14.56^****^
ROM (° ± S.D)	92.0 ± 24.08	119.3 ± 8.86^****^
Phenotype		
I (%)	5 (3.6)	15 (10.8)
II (%)	23 (16.5)	27 (19.4)
III (%)	64 (46.0)	63 (45.3)
IV (%)	35 (25.2)	25 (18.)
V (%)	12 (8.6)	9 (6.4)

**** *P* < 0.001

**Fig. 5:**
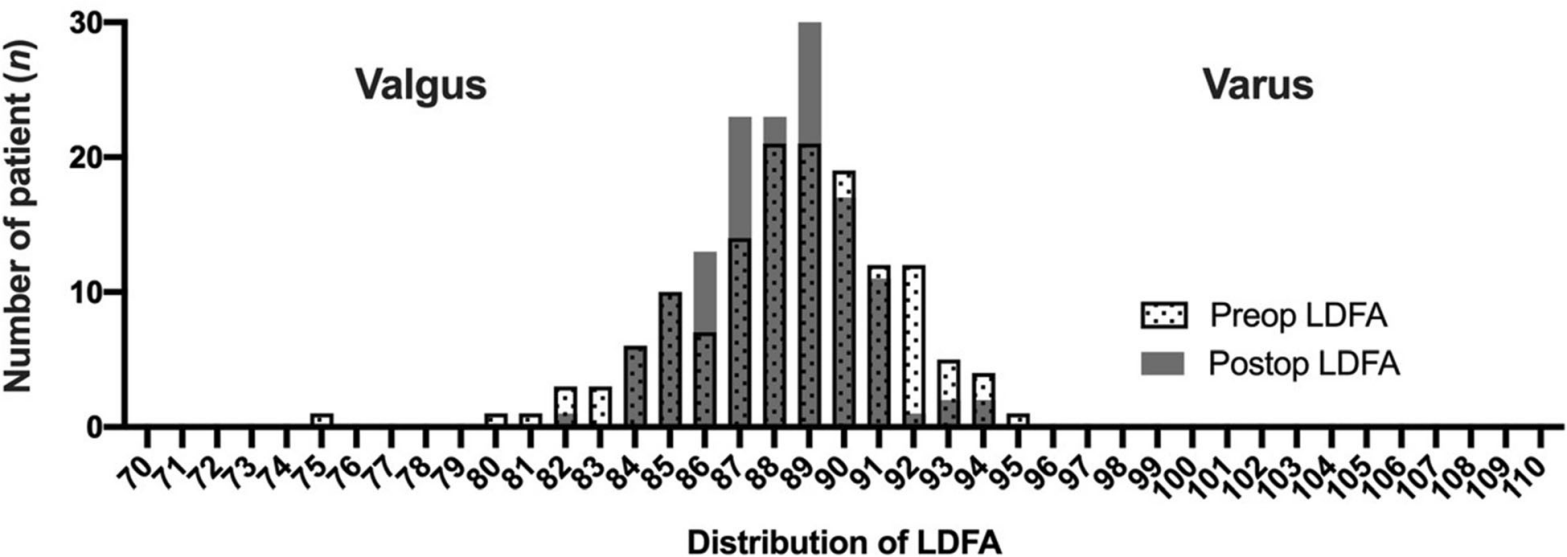
Distribution of preoperative and postoperative LDFA

**Fig. 6:**
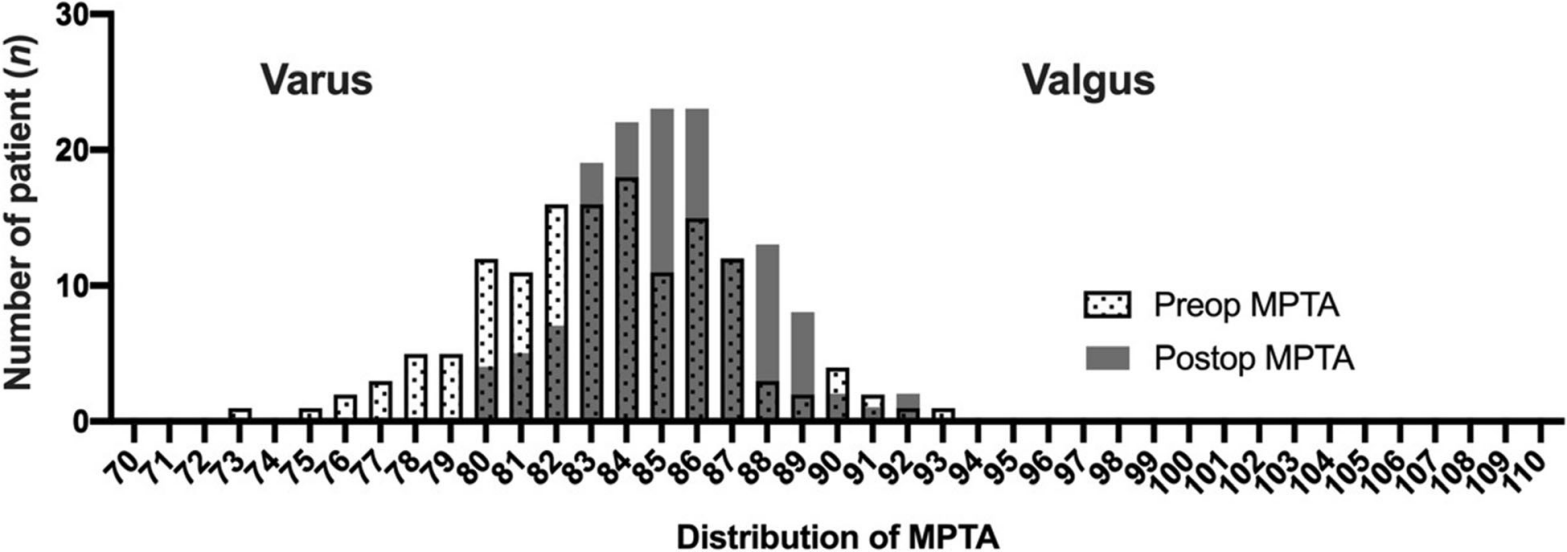
Distribution of preoperative and postoperative MPTA

Using the phenotype-oriented KA method, the postoperative distribution of the LDFA converged to 86° to 90° (Fig. 5) and the postoperative distribution of the MPTA converged to 84° to 86° (Fig. 6). In contrast to the MPTA, the postoperative LDFA had no significant difference compared to the preoperative LDFA (Table 1 and Fig. 5).

The functional and radiographic outcomes of each phenotype of knee are shown in Tables 2, 3, 4, 5 and 6. Significant improvement in functional outcomes was observed in all five knee phenotypes. For the type 1 knee, no significant radiographic change was noted between preoperative and postoperative parameters (Table 2). For the type 2 knee, no significant radiographic change except a significant improvement in HKAA(*P* = 0.04) was observed (Table 3). For type 3 knee, significant changes were found in all radiographic parameters except in LDFA. Though residual postoperative varus existed in MPTA, the postoperative joint line remained relatively parallel to the ground (TJLA = 1.31°) (Table 4). For type 4 knee, significant changes were noted in all radiographic parameters including the LDFA. Residual postoperative varus existed in MPTA, but the postoperative joint line also remained relatively parallel to the ground (TJLA = 1.29°) (Table 5). For the type 5 knee, significant changes were noted in all radiographic parameters except in MPTA and TJLA. The valgus alignment got significant correction compared to preoperative alignment (Table 6).

**Table 2:** Preoperative and postoperative data of type 1 knee

(*n* = 5)	Pre-operative	Post-operative	*p*-value
HKAA	2.9 ± 4.82	−0.8 ± 1.58	0.18
AA	5.7 ± 2.32		
KAA	−2.2 ± 4.58	−5.5 ± 2.21	0.19
TJLA	1.8 ± 2.13	1.4 ± 1.74	0.80
LDFA	88.7 ± 1.71	87.0 ± 1.05	0.23
MPTA	87.6 ± 0.67	88.2 ± 1.03	0.17
OKS	15.6 ± 8.11	45.40 ± 2.19	**< 0.01**
CKSS	91.8 ± 24.24	176.8 ± 18.02	**< 0.01**
ROM	91.4 ± 30.41	122.4 ± 3.85	0.11

**Table 3:** Preoperative and postoperative data of type 2 knee

(*n* = 23)	Pre-operative	Post-operative	*p*-value
HKAA	3.7 ± 7.07	1.2 ± 2.11	**0.04**
AA	5.4 ± 1.32		
KAA	−2.0 ± 6.67	−4.4 ± 2.50	0.11
TJLA	1.2 ± 3.68	1.2 ± 1.58	0.94
LDFA	85.0 ± 3.20	85.9 ± 1.84	0.15
MPTA	84.4 ± 2.33	85.3 ± 2.18	0.08
OKS	17.8 ± 8.22	44.6 ± 2.57	**< 0.01**
CKSS	91.7 ± 33.68	172.7 ± 13.23	**< 0.01**
ROM	93.7 ± 25.11	117.6 ± 10.67	**< 0.01**

**Table 4:** Preoperative and postoperative data of type 3 knee

(*n* = 65)	Pre-operative	Post-operative	*p*-value
HKAA	11.83 ± 5.13	4.40 ± 2.58	**< 0.0001**
AA	7.26 ± 1.69		
KAA	2.65 ± 4.56	−4.35 ± 2.45	**< 0.0001**
TJLA	2.86 ± 2.49	1.31 ± 1.06	**< 0.0001**
LDFA	88.61 ± 1.20	88.26 ± 1.48	0.0735
MPTA	82.03 ± 3.08	84.57 ± 2.22	**< 0.0001**
OKS	17.89 ± 7.76	43.74 ± 3.73	**< 0.0001**
KSFS	86.40 ± 32.43	170.6 ± 16.57	**< 0.0001**
ROM	91.29 ± 24.72	119.5 ± 8.35	**< 0.0001**

**Table 5:** Preoperative and postoperative data of Type 4 knee

(*n* = 34)	Pre-op of Type 4	Post-op of Type 4	*p*-value
HKAA	14.09 ± 4.14	5.63 ± 2.97	**< 0.0001**
AA	8.41 ± 1.71		
KAA	3.19 ± 3.88	−3.92 ± 2.75	**< 0.0001**
TJLA	4.22 ± 2.67	1.29 ± 1.24	**< 0.0001**
LDFA	91.66 ± 1.39	90.01 ± 1.72	**< 0.0001**
MPTA	82.42 ± 2.81	84.69 ± 1.91	**< 0.0001**
OKS	18.25 ± 6.80	44.33 ± 3.13	**< 0.0001**
KSFS	97.31 ± 24.33	173.9 ± 11.27	**< 0.0001**
ROM	93.00 ± 22.84	120.5 ± 8.75	**< 0.0001**

**Table 6:** Preoperative and postoperative data of type 5 knee

(*n* = 12)	Pre-operative	Post-operative	*p*-value
HKAA	−9.64 ± 4.04	−2.88 ± 2.80	**0.0012**
AA	4.21 ± 1.67		
KAA	−12.46 ± 3.39	−6.61 ± 1.90	**0.0004**
TJLA	−2.75 ± 4.12	−2.20 ± 3.30	0.5297
LDFA	83.22 ± 1.35	86.65 ± 1.94	**0.0002**
MPTA	89.39 ± 1.98	89.14 ± 1.90	0.6799
OKS	14.00 ± 8.49	43.08 ± 3.06	**< 0.0001**
KSFS	85.75 ± 33.89	171.3 ± 13.85	**< 0.0001**
ROM	89.58 ± 23.40	116.4 ± 9.62	**0.0012**

Table 7 shows the comparison of postoperative knee alignment parameters and functional outcomes between the five knee phenotypes. Though significant difference was noted in postoperative knee alignment parameters between the five knee phenotypes, no significant difference was found in functional outcomes including OKS, CKSS and ROM between the five knee phenotypes.

**Table 7:** Comparison of postoperative radiographic and functional outcomes among five knee phenotypes after the KA-TKA

Parameters	Knee Phenotype					*P* value
	1	2	3	4	5	
HKAA (° ± S.D)	−0.8 ± 1.58	1.2 ± 2.11	4.4 ± 2.58	5.6 ± 2.97	−2.9 ± 2.80	**< 0.001**
LDFA (° ± S.D)	87.0 ± 1.05	85.9 ± 1.84	88.3 ± 1.48	90.0 ± 1.72	86.7 ± 1.94	**< 0.001**
MPTA (° ± S.D)	88.2 ± 1.03	85.3 ± 2.18	84.6 ± 2.22	84.7 ± 1.91	89.1 ± 1.90	**< 0.001**
KAA (° ± S.D)	−5.45 ± 2.21	−4.43 ± 2.50	−4.35 ± 2.45	−3.92 ± 2.75	−6.61 ± 1.90	**0.03**
TJLA (° ± S.D)	1.44 ± 1.74	1.22 ± 1.58	1.31 ± 1.06	1.29 ± 1.24	−2.20 ± 3.30	**< 0.001**
OKS (Points ± S.D)	45.4 ± 2.19	44.6 ± 2.57	43.7 ± 3.73	44.3 ± 3.13	43.1 ± 3.06	0.570
CKSS (Points ± S.D)	176.8 ± 18.02	172.7 ± 13.23	170.6 ± 16.57	173.9 ± 11.27	171.3 ± 13.85	0.809
ROM (° ± S.D)	122.4 ± 3.85	117.6 ± 10.67	119.5 ± 8.35	120.5 ± 8.75	116.4 ± 9.62	0.529

## Discussion

The aim of this study was to investigate the distribution of the five phenotypes in advanced OA population and the clinical outcomes of this phenotype-oriented KA-TKA. The results showed that 71.2% of advanced OA were varus knee, which comprised either tibial varus (type 3) or both tibial and femoral varus (type 4) phenotype, 20.1% were neutral knee, which comprised transverse joint line (type 1) and oblique joint line (type 2) phenotype, 8.6% were valgus knee (type 5) phenotype. With individualized alignment targets for the KA method, all five knee phenotypes had significantly improved functional outcomes and a total survival rate of 99.3% in the 3-year follow-up period.

The distribution of knee phenotype in the advanced OA population was different from that in the non-arthritic population. In the non-arthritic population, constitutional varus accounted for 20–30% [9, 12]. In the present study, 71.2% of varus alignment phenotypes were observed in the advanced OA population. In the advanced OA population, the ratio of varus phenotypes was obviously higher than that in the non-arthritis population. The significant increased ratio of varus alignment phenotypes may contributed to the higher incidence of advanced OA knee in population with constitutional varus knee. A report by Sharma et al. showed that non-arthritis patients with varus alignment had a significantly higher risk of onset of OA in a 30-month period of observation [13]. Higano et al. also noted that patients with constitutional varus knee had a significant higher risk than non-varus knee progressing to advanced OA knee in a 20-year prospective observational study [14]. These findings suggested that in most cases, advanced varus OA knee were progressed from constitutional varus knee rather than from neutral-aligned knee. Restoring the advanced varus arthritic knee to pre-arthritic constitutional varus knee may decrease soft tissue adjustment and preserve normal knee kinematics [15]. KA-TKA according to original knee phenotype allows simultaneous improvement in surgical outcomes and avoidance of extreme malalignment that may compromise long-term outcomes.

The average postoperative MPTA in our study was 85.2 ± 2.47°, which was smaller than in previous reports of KA-TKA [2, 16]. However, we included 34 patients with both varus alignment of femur and tibia (type 4 knee), which were excluded in previous similar studies, that may account for the lower MPTA in our study. The varus inclination of the tibial component may be a concern for long term survival of the prosthesis and early loosening [17]. However, good functional outcome and a 10-year implant survival rate of 97.5% have been reported for varus outlier of tibial alignment [8, 18]. Recent studies further indicated that the main causes of tibial component failure were posterior subsidence, posterior edge wear, and varus femur component, rather than medial subsidence or tibia varus [19, 20].

The average LDFA of our study was 88.1 ± 2.14°, which is similar to the findings in previous studies: The LDFA is 1.3 to 2° more valgus than that of MA-TKA [21–23]. The preserved valgus orientation of the distal femur counteracts the varus orientation of the tibia and balances the load to the medial and lateral compartment, which creates a parallel joint line to the ground though residual varus of tibia is present [24, 25].

In previous randomized trials comparing the KA with the MA method, the postoperative OKS, CKSS, and ROM ranged from 40 to 42 points, 160–190 points and 119–121° in KA group, respectively. Except a randomized study which used computer navigation in the MA group [21], KA-TKA performed with PSI had overall better functional outcome than MA-TKA [21–23]. In our protocol, the postoperative OKS, combined KSS and ROM were 44.0 ± 3.31 points, 172.1 ± 14.56 points and 119.3 ± 8.86°, respectively, which were comparable with that of previous KA-TKA studies. These findings suggest that this phenotype-oriented KA-TKA is a practical and reliable method for KA-TKA especially when PSI or computer navigation is not available.

Using our protocol, similar great functional improvement were observed in all patients, though significant difference were found in postoperative alignment parameters between the five knee phenotypes. This finding supports the rationality of setting individualized alignment target according to original knee phenotype. This is the first study to evaluate the surgical outcomes of TKA performed using the KA method without navigation or PSI. By determining the preoperative knee phenotype, setting alignment targets, and using the bone cut thickness as a check reference, it was possible to perform TKA as planned. Our data showed that this phenotype-oriented KA method was a safe and accurate approach for TKA with generic instruments. This method enabled preoperative-planned alignment between the original anatomy and mechanical alignment, while avoiding excessive soft tissue release and preserving the original knee kinetics [26, 27]. In this study, we provide target alignment angles for each type of OA knee and detailed surgical techniques. Our results showed that this approach had excellent functional outcomes and a 99.3% implant survival rate over a 3-year period. We recommend the phenotype-oriented KA method when PSI is not available or for cases with type 4 knee (both femur and tibia varus).

This study has a number of limitations. First, the study had no MA group as a control to compare functional and radiographic outcomes. However, compared to the results of previous randomized control comparing the KA with the MA method, we found that the radiographic and functional outcomes were comparable with that of KA-TKA performed with PSI, which was superior to the MA group [21–23]. Second, the three-year follow-up time was relatively short to assess long-term complications such as aseptic loosening, which may be affected by component alignment [4, 28]. Although positive 10-year results of KA-TKA have been published [8]. The long term survivorship of varus tibial component in type 3, and type 4 knee phenotypes may be account for the ground-parallel joint line. Third, the number of cases were relatively small. Further studies with a larger population should be done to confirm these results.

## Data Availability

The datasets used and analyzed during the current study are available from the corresponding author on reasonable request.

## Abbreviations

OA: Osteoarthritis
TKA: Total knee arthroplasty
MA: Mechanical alignment
KA: Kinematic alignment
PSI: Patient specific instrument
HKAA: Hip-knee-ankle angle
KAA: Knee alignment angle
TJLA: Tibial joint line obliquity angle
LDFA: Lateral distal femur angle
MPTA: Medial proximal tibial angle
AA: Angle between the mechanical axis of femur and anatomical axis
MCL: Medial collateral ligament
